# Selective emodin toxicity in cancer cells

**DOI:** 10.18632/oncotarget.16611

**Published:** 2017-03-27

**Authors:** Panagiota Kolitsida, Hagai Abeliovich

**Affiliations:** Department of Biochemistry and Food Science, Hebrew University of Jerusalem, Rehovot, Israel

**Keywords:** mitochondria, respiration, emodin, superoxide, cancer

Emodin is an anthraquinone extracted from plant tissue which has established anti-cancer, anti-inflammatory and anti-diabetic effects [1, 2]. Numerous directed studies have indicated that emodin has a plethora of effects on diverse cellular processes. In this issue, groundbreaking new work by Dumit and colleagues [3] present the first unbiased molecular characterization of emodin's mechanism of action. Using affinity chromatography coupled with SILAC-based mass spectrometry, the authors identified 10 emodin-binding proteins from CaCo-2 cell extracts. Strikingly, three of these proteins turn out to be NADPH- dependent oxidoreductases. In agreement with this, they report that emodine treatment has dramatic effects on the cellular NADPH/NADP^+^ ratio. In parallel, unbiased proteomic screens for proteins whose levels are affected by emodin treatment, the authors find that the major effect of emodine is on components of the mitochondrial electron transport chain. They then turn their attention to try to understand why emodine is selectively toxic to cancer cells. They find that mitochondrial complex I proteins, in agreement with the MS data, are strongly down-regulated upon emodine treatment of cancer cells. However the effect is specific to cancer-derived cell lines: lines derived from quiescent cells show a much reduced effect of emodin on complex I components. In accordance with a mitochondria-centric effect of emodin on cells, pretreatment of quiescent cell lines with doxycycline, which targets mitochondrial protein synthesis apparatus, led to an increased effect of emodin in these cells and to a phenocopy of the transformed cell line sensitivities. The increased sensitivity of cancer cells to emodin correlates with the Warburg effect, a phenomenon known since the 1920s, wherein many cancer cell types shut down oxidative phosphorylation in favor of glycolysis [4]. Indeed, robust respiratory capacity seems to differentiate between emodin sensitivity and resistance since hampering mitochondrial function of normal cells by chemical intervention with tetracyclin-family antibiotics, which selectively target the mitochondrial translation machinery, shows a striking synergy with emodin treatment of non-cancer cells. Much like cancer cells, the baker's yeast, *Saccharomyces cerevisiae*, shuts down oxidative phosphorylation in the presence of glucose and produces ethanol as a terminal metabolic product under these conditions, a phenomenon known as the Crabtree effect [5]. Remarkably, yeast cells, just like mammalian cells, show an emodin sensitivity that is correlated with the activity level of oxidative phosphorylation. Since the ROS scavenging agent NAC can counteract the toxic effects of emodin, the authors suggest that one aspect of emodin toxicity is the increase in mitochondrial ROS generation.

The quinone group, in its ionized form, could hypothetically transfer protons across membranes. Thus emodin could act as an uncoupling agent. Indeed, addition of emodine to isolated respiring mitochondrial preparations indicate that this is indeed the case. In fact, the uncoupling effect of emodin on cancer cell lines is greater than it is on quiescent cells. Importantly however, emodin must have additional effects beyond uncoupling, since the classic mitochondrial uncoupler CCCP does not exhibit selective activity towards cancer cell lines, in contrast with emodin. Several findings in this paper support the idea that emodin is not simply an uncoupler: First, its global effects on mitochondrial gene expression and its ability to directly bind oxidoreductases are not classical or definitive characteristic of uncoupling agents. Second, the authors show that classic uncouplers do not mimic the selective effect of emodin on cancer cells, which may relate to its specific binding to redox factors.

In mammalian cells, the main site of superoxide production is complex I, where accumulation of reduced flavin leads to a slow side reaction with molecular oxygen to generate superoxide anion [6, 7]. The potential similarity and chemistry of emodin to ubiquinone may suggest the Q site of complex I as one possible target for emodin. However the further finding by Dumit et al that yeast cells, which lack complex I altogether, show a similar response to emodin, may suggest a more subtle mechanism involving complex III, as well as additional sources of superoxide production. This observation is also consistent with the observed decrease in complex I levels upon emodin treatment of mammalian cells, since it is unlikely (but not impossible) that emodin decreases complex I levels and simultaneously increases superoxide production from complex I.

To summarize, the pioneering work by Dumit et al clearly indicates that the main target of emodin in cells is the mitochondrion. This implies that much of the wide spectrum of molecular effects previously documented in cells upon emodin treatment must be secondary to its effect on mitochondria. On the other hand, even within mitochondria, the effects of emodin seem to be multifaceted; it simultaneously causes uncoupling, which should lower superoxide production due to increased oxidation of the FMN component in complex I [8], but at the same time it causes an increase in superoxide anion production. The resolution of these points remains the topic of future research.
